# Detection of Zoonotic *Cryptosporidium ubiquitum* in Alpine Wild Ruminants

**DOI:** 10.3390/pathogens10060655

**Published:** 2021-05-25

**Authors:** Tiziana Trogu, Nicoletta Formenti, Marianna Marangi, Roberto Viganò, Radames Bionda, Annunziata Giangaspero, Paolo Lanfranchi, Nicola Ferrari

**Affiliations:** 1 Istituto Zooprofilattico Sperimentale della Lombardia e dell’Emilia Romagna ‘‘Bruno Ubertini’’ (IZSLER), via Bianchi 7/9, 25124 Brescia, Italy; tiziana.trogu@gmail.com; 2 Dipartimento di Scienze Agrarie, degli Alimenti e dell’Ambiente, Università degli Studi di Foggia, via Napoli 25, 71121 Foggia, Italy; marianna.marangi@unifg.it (M.M.); annunziata.giangaspero@unifg.it (A.G.); 3 Studio Associato AlpVet, Piazza Venzaghi 2, 21052 Busto Arsizio, Italy; r.vigano@alpvet.it; 4 Ente di Gestione delle Aree Protette dell’Ossola, Villa Gentinetta-Viale Pieri 27, 28868 Varzo, Italy; rada.bionda@libero.it; 5 Dipartimento di Medicina Veterinaria, Università degli Studi di Milano, via dell’Università 6, 26900 Lodi, Italy; paolo.lanfranchi@unimi.it (P.L.); nicola.ferrari@unimi.it (N.F.); 6 Centro di Ricerca Coordinata Epidemiologia e Sorveglianza Molecolare delle Infezioni, EpiSoMI, Università degli Studi di Milano, 20133 Milan, Italy

**Keywords:** *Cryptosporidium* spp., *Cryptosporidium ubiquitum*, wild ruminants, Alps

## Abstract

*Cryptosporidium* is a widespread apicomplexan protozoan of major zoonotic importance, characterized by a wide host range, and with relevant economic implications and potential negative effects on livestock and wildlife population dynamics. Considering the recent strong demographic increase of alpine ungulates, in this study, carried out in the Italian Northwestern Alps, we investigated the occurrence of *Cryptosporidium* spp. in these species and their potential involvement in environmental contamination with *Cryptosporidium* spp. oocysts. The immune-enzymatic approach revealed a *Cryptosporidium* prevalence of 1.7% (5/293), 0.5% (1/196) and 3.4% (4/119) in alpine chamois (*Rupicapra rupicapra*), red deer (*Cervus elaphus*) and roe deer (*Capreolus capreolus*), respectively. Positive samples were subjected to polymerase chain reaction (PCR) amplification for the COWP and gp60 genes. The presence of *Cryptosporidium* was confirmed in one chamois and four roe deer. Sequences obtained clustered within *Cryptosporidium* *ubiquitum*, currently recognized as an emerging zoonotic species. This finding represents the first detection of zoonotic *C.* *ubiquitum* in chamois and in the Alpine environment. Despite the low observed prevalences, environmental contamination by oocysts could play a role as a potential source of infections for humans and livestock.

## 1. Introduction

Parasites naturally develop strategies to increase their spread in the environment. Some of them infect only one or a few target species [1], while others have a broad spectrum of hosts leading to a wider spread [1,2]. Many generalist parasites can infect both humans and animals, thus being potentially responsible for emerging infectious disease outbreaks [2]. Some *Cryptosporidium* species represent an emblematic example of the latter category, globally causing major enteric diseases in humans [3] and in a wide range of animals, including both livestock and wildlife [4,5,6].

These apicomplexan protozoans are highly successful parasites: their direct life cycle (oro-fecal route), the high number of oocysts released with feces and the long-lasting survival of oocysts in water and soil [7,8] lead to a high environmental spread and contamination. Moreover, the low infective dose [9] and the oocysts’ ability to resist routine wastewater treatment and common disinfectants [10,11,12,13] increase the risk for human infection, representing a major public health concern. These parasites are indeed a major cause of severe foodborne and waterborne outbreaks [6]. Over the years, the number of recognized species within the *Cryptosporidium* genus has gradually increased [14]. Among zoonotic species, *C. parvum* is the main agent responsible for human infection, although in recent years, *C. ubiquitum* (formerly known as the cervine genotype) has been emerging as a new major zoonotic species, especially in industrialized countries [15]. This species is able to affect a wide range of domestic and wild animals [10,16,17,18,19] and these hosts could be involved in its spread and environmental contamination increasing the potential for human exposure [4]. In humans, *Cryptosporidium* can severely affect immunocompromised adults and children, with longer-term consequences for malnourished individuals, such as growth stunting and cognitive deficits [20]. Vaccines are not currently available and drug treatment options are limited and do not provide a reliable strategy for infection control [20,21].

In adult domestic ruminants, *Cryptosporidium* is often asymptomatic. In young subjects, clinical signs can range from mild diarrhea and growth retardation to death, depending on the parasite species and burden and on the susceptibility, age and health status of the host [10,22], leading to reduced productivity and important economic losses [10,23,24].

While extensive knowledge on the effects and spread of *Cryptosporidium* in humans and domestic ruminants is available, its impact and circulation in wildlife are still poorly known, partly due to the intrinsic challenges related to field sampling. In the Alps, except for a sporadic report in Alpine Ibex (*Capra ibex*) in Switzerland [25], *Cryptosporidium* infection in wild ruminants has not been investigated and the epidemiological role of these host species is still unknown. Therefore, considering that zoonotic species of *Cryptosporidium* (i.e., *Cryptosporidium. parvum* and *Cryptosporidium ubiquitum*) are able to affect both domestic and wild ruminants [16,26,27], the present study aimed at investigating the presence of *Cryptosporidium* in the alpine environment. Among the ungulate species present in the alpine areas, we focused on chamois (*Rupicapra rupicapra*), red deer (*Cervus elaphus*) and roe deer (*Capreolus capreolus*), which are hunted species. Thus, the potential zoonotic risk should be evaluated also in terms of the amount of game meat harvested every year [28], especially in relation to evisceration and handling phases, which certainly represent crucial moments for the exposure to infection.

## 2. Results

Sampling was carried out within two contiguous territories, a protected area and a hunting district in Piedmont Italian Alps. Overall, five chamois were positive to *Cryptosporidium*, with an observed prevalence of 1.7% (confidence interval (CI) 95%: 0.7–3.9). The positive animals were two young and three adults. Additionally, in the hunting district one red deer kid, and four roe deer (two kids, one yearling and one adult) were positive, with prevalences of 0.5% (CI 95%: 0–2.8) and 3.4% (CI 95%: 1.3–8.3), respectively. In the protected area, all red deer and roe deer were negative to the immuno-enzymatic test. Results are summarised in Table 1.

No cases of diarrhea nor evident clinical signs were observed, either in animals sampled in the protected area or in those monitored in the hunting district.

The positivity to *Cryptosporidium* was confirmed by molecular analyses in five (one adult chamois and four roe deer) out of the 10 samples positive to immune-enzymatic tests. Samples from the chamois and from two of the roe deer resulted positive to both *COWP* and *gp60* polymerase chain reaction (PCR) protocols. All the five obtained sequences, identical to each other, clustered within the *C. ubiquitum* group, showing a 100% identity with *C. ubiquitum* sequences available in GenBank.

## 3. Discussion

To the best of our knowledge, this study represents the first report of *Cryptosporidium* spp. in chamois, as well as its first detection in roe deer and red deer from the alpine region, confirming the peculiarity of this generalist protozoan which is able to colonize several host species and to survive in different and often extreme environments. In addition, the species *C. ubiquitum* was detected in both chamois and roe deer, raising potential concerns related to its zoonotic potential.

Immune-enzymatic tests showed a prevalence of 1.7% in chamois, 0.5% in red deer and 3.4% in roe deer, highlighting the susceptibility of all the three species and their role as *Cryptosporidium* hosts. However, no animals with *Cryptosporidium* symptoms (diarrhea) have been detected during the sampling. Not all the positive samples from enzyme-linked immunosorbent assay (ELISA) tests were subsequently confirmed by PCR analyses. Impurities and materials (e.g., complex polysaccharides, bilirubin, and bile salts) in fecal samples may affect the performance of both extraction and PCR [29,30], as reported by other studies comparing immune-fluorescence and immune-enzymatic methods with molecular assays [31,32].

*Cryptosporidium ubiquitum* was detected in different countries in cervids such as white-tailed deer (*Odocoileus virginianus*) [16], roe deer [14], red deer [27] and sika deer (*Cervus nippon*) [26], highlighting the deer’s importance as a potential source of zoonotic cryptosporidiosis in the environment [33]. Moreover, this parasite species was also identified in mouflon (*Ovis aries musimon*) [34] and alpine ibex [32]. Indeed, in several studies *C. ubiquitum* and *C. parvum* are reported as the main species found in wild ungulates in Europe [27,34,35,36].

In the present study, prevalences and recorded species are consistent with data recorded in wild ungulates in other European countries [36,37]. In our case, the low recorded prevalence might be related to the period of sampling. In ruminants, the most critical phases for *Cryptosporidium* infection are the first weeks of life [38], when, in addition to the higher susceptibility of calves and kids to the infection, an increase in oocyst excretion by mothers is commonly recorded [39,40]. In mountain wild ruminants this period corresponds to late spring, but adverse weather condition and the persistent snow until early summer did not allow us to obtain fresh fecal samples in the first weeks of life, especially for chamois, likely missing the peak of oocyst shedding. Moreover, autumnal sampling linked to hunting activities has been carried out exclusively on animals of at least 5–6 months of age. Thus, the prevalence found in the present study is likely an underestimation of the actual prevalence of infection. The Alps represent a complex environment, characterized by a strong human presence related to traditional zootechnical activities, and to the increasing development of new outdoor activities. Sporadic cryptosporidiosis cases linked to outdoor activities have indeed been recently observed [41,42].

Regardless of the quantitative role of these ungulates species as a source of environmental infection, these results highlight their susceptibility to infection. Indeed, the higher prevalence recorded in roe deer, usually living in more anthropized areas, could support the role of livestock or human outdoor activities as a source of infection.

On the other hand, we detected also several positive chamois, in spite of them living in more extreme habitats with lower contact with humans and livestock. This suggests a potential transmission from other host species or the exposure to some environmental sources of infection. In this regard, it should be considered that chamois usually share pastures with alpine ibex, a species susceptible in turn to *Cryptosporidium* infection [25,32]. Despite the low prevalence recorded, the role of wildlife within the life cycle of a generalist parasite such as *Cryptosporidium* should not be underestimated as free-living populations can be considered as indicators of the level of environmental contamination. Indeed, considering that for *Cryptosporidium* the infectious dose is very low, it is possible that the analyzed species could play a role as maintenance hosts of *Cryptosporidium* infection even in extreme, scarcely anthropized habitats. Therefore, these results should be considered in light of the high abundance of host species reached in the Alps [43], and the consequent spatial overlap with humans and domestic animals. In this regard, the health risks for domestic animals at shared pastures should be taken into account due to *Cryptosporidium*-induced potential production losses and economic impacts. In addition, we must consider that wild ruminant grazing areas are usually located near watersheds that often provide municipal drinking water. Therefore, potential health risks related to the resistance of the protozoan and the lack of specific treatments should be taken into consideration for environmental contamination and subsequent human infection [44].

In conclusion, this study shows the presence of *Cryptosporidium* in alpine wild ruminants. In particular, this represents the first record of the presence of zoonotic *C. ubiquitum* in chamois and roe deer. Further and broader investigations are ongoing to better clarify the role of these host species into *Cryptosporidium* circulation in extreme Alpine environments and the potential sanitary risks connected to it.

## 4. Materials and Methods

### 4.1. Study Area

The study area is located in the northeastern part of Piedmont region, in the Lepontine Alps (Italy) (46°07′ N, 8°17′ E), close to the border with Switzerland (Canton of Valais and Canton of Tessin). The field sampling has been carried out in two contiguous territories: (i) Alpe Devero protected area, that has a total extent of 5.454 ha within Alpe Veglia-Alpe Devero Natural Park, having an altitude ranging from 1.600 to 3.235 m a.s.l. In this area hunting is banned and monitoring was carried out on living, free-ranging animals. (ii) Verbano Cusio-Ossola province hunting district (VCO 2-Ossola Nord) with an extension of 72.740 ha, ranging from 250 to 3.373 m a.s.l., where hunted animals were investigated post-mortem. In the protected area, alpine ibex (*Capra ibex*) is the most represented wild ungulate species, with a density of 2.2 subjects/sq. km; chamois are 1.9 subjects/sq. km; red deer are about 1.2 subject/sq. km, while roe deer show a limited presence. In the hunting district, densities were estimated at 6.7 chamois/sq. km, 2 red deer/sq. km and 2.5 roe deer/sq. km. Traditional livestock farming, with summer pastures, is present throughout the study area, with a population of about 6.4 bovines/sq. km, 11.5 sheep/sq. km and 14.1 goats/sq. km, sharing grazing and water sources with wildlife. Human outdoor activities are consistent throughout the year, with the presence of about 2.5 million tourists/year in Verbano-Cusio-Ossola province.

### 4.2. Sampling

During a three-year campaign, a total of 222 fecal samples were collected in the protected area. Transects were set up monthly from June till November to monitor the animals. Ruminants were localized and observed by 10 × 50 binoculars and a 60× telescope in order to record the species, and, when possible, age and gender, and to monitor the occurrence of clinical symptoms (e.g., body score condition, fur quality, correct molt). Once abandoned by ruminants, the pasture was reached to collect fresh fecal samples from the observed groups, even if it was not possible to determine the exact correspondence between each fecal sample and the specific individual animal. Since for animals older than one year, exact age cannot be estimated from feces morphology, animals were grouped in two age classes: kids (0–6 months old), and adults. Fresh fecal samples of an overall 126 chamois (47 kids, 79 adults), 90 red deer (19 kids, 71 adults) and 6 roe deer (2 kids, 4 adults) were collected from the ground, using a sterile disposal latex glove and then placed individually into a disposable plastic bag. The specimens were transported to the laboratory in refrigerated conditions at 4 °C.

In the hunting area, hunting activity is carried out from September to November in accordance with the Italian law (Law n. 157 of 11/02/1992) and hunters need to carry culled ungulates to game control centers. At control centers, age, sex, shooting site (valley, altitude, exposure, etc.) of each animal are registered. Although here age classes of animals can be defined more precisely compared to field conditions, for consistency with the previous sampling animals were classified only into young and adults. We collected directly from the rectal ampoule 167 samples from chamois (12 kids, 155 adults), 106 from red deer (35 kids, 71 adults) and 113 from roe deer (27 kids, 86 adults).

In both cases, feces’ consistency was noted and samples were split in two parts: one stored at −20 °C for immune-enzymatic analyses and the other one preserved in potassium dichromate solution (2.5%) at 4 °C until molecular analysis [45].

### 4.3. Immuno-Enzymatic and Molecular Analyses

A commercial immuno-enzymatic kit (RIDASCREEN^®®^ Cryptosporidium; R-Biopharm AG, Germany) has been used according to the manufacturer’s recommendations.

Genomic DNA was extracted from 200 mg of fecal samples previously positive to the immuno-enzymatic test. Fecal samples were subjected to three cycles of sonication and, after addition of proteinase K, incubated overnight at 56 °C. Then, DNA was extracted from individual samples using QIAamp DNA Stool Mini Kit (Qiagen, Milan, Italy) according to the manufacturer’s recommendations, and stored at −20 °C until molecular analysis.

Two PCR protocols were performed: a semi-nested PCR targeting the COWP gene and a nested PCR, targeting the gp60 gene. A fragment within the COWP gene of about 360 bp was amplified by using primers CRY15D (5’-GTAGATAATGGAAGRGAYTGTG-3’) and CRY9D (5’-GGACKG AAATRCAGGCATTATCYTG-3’) for the first PCR and primer CRYINT2D (5’-TTTGTTGAAGARGGAAATAGATGTG-3’) with primer CRY9D for the semi-nested PCR [46]. In order to obtain a more robust information, a fragment within gp60 gene of about 400 bp was also amplified by using primers AL3531 (5-ATAGTCTCCGCTGTATTC-3′) and AL3533 (5′-GAGATATATCTTGGTGCG-3′) for the first PCR and primers LX0029 (5′-TCCGCTGTATTCTCAGCC-3′) and AL3532 (5′-TCCGCTGTATTCTCAGCC-3′) for the nested PCR [47]. Both PCRs were carried out in 25 μL of standard buffer, containing thermostable polymerase and dNTPs (Ready Mix REDTaq, Sigma, St. Louis, MO, USA) plus 100 pmol of each primer. Approximately 100 ng of genomic DNA was incorporated into each reaction, and a negative control sample (no-template) and a known positive control sample (previously found positive) were included in each PCR run. The amplifications were carried out using the following cycling protocol: 95 °C/3 min (initial denaturation), followed by 35 cycles of 94 °C/45 s (denaturation), 50 °C (first PCR) or 51 °C (nested PCR) for 45 s (annealing) and 72 °C/1 min (extension), with a final extension of 72 °C/4 min. The PCR products were run on 1.2% agarose gel and positive samples were purified with Exonuclease I (EXO I) and Thermosensitive Alkaline Phosphatase (FAST AP) (Fermentas, Waltham, MA, USA) enzymes, according to the manufacturer’s instructions. The obtained PCR fragments were directly sequenced in both directions using the ABI PRIMS BygDye Terminator v. 3.1Cycle Sequencing Kit (Applied Biosystems, Foster City, CA, USA) with the same primers as the corresponding PCRs reaction, according to the manufacturer’s instructions. The obtained sequences were determined on an ABI PRISM 3130 Genetic Analyzer (Applied Biosystems, USA), and chromatograms were visually inspected using the Finch TV software to remove primer regions and bad-quality regions. Once the sequences had been cleaned up, each sequence was compared with the *Cryptosporidium* nucleotide sequences available in GenBank databases using BLAST NCBI (2020).

## Figures and Tables

**Table 1:** Prevalence (P (%)–CI 95%) of *Cryptosporidium* in chamois, red deer and roe deer, in different years within protected area and hunting district. Nr = total number of samples collected; Pos = number of samples positive to immuno-enzymatic test.

		Chamois			Red Deer			Roe Deer		
Campaign	Area	Nr	Pos	P (%) CI 95%	Nr	Pos	P (%) CI 95%	Nr	Pos	P (%) CI 95%
First	Protected area	47	2	4.25% (1.17–14.2)	-	-	-	-	-	-
Second	Protected area	55	2*	3.63% (1–12.3)	70	0	0%	6	0	0%
	Hunting district	91	1	1.09% (0.1–5.9)	53	0	0%	64	3*	4.68% (1.6–12.9)
Third	Protected area	24	0	0%	20	0	0%	-	-	-
	Hunting district	76	0	0%	53	1	1.88% (0.3–9.94)	49	1 *	2.04% (0.3–10.6)
Total		293	5	1.70% (0.7–3.9)	196	1	0.51% (0–2.8)	119	4	3.36% (1.3–8.3)

* Samples tested PCR-positive to *Cryptosporidium*: one chamois in the protected area and four roe deer in the hunting district.

## Data Availability

Data generated or analysed during this study are included in the published article.
